# Research trends in contemporary health economics: a scientometric analysis on collective content of specialty journals

**DOI:** 10.1186/s13561-023-00471-6

**Published:** 2024-01-25

**Authors:** Clara C. Zwack, Milad Haghani, Esther W. de Bekker-Grob

**Affiliations:** 1 Department of Nursing and Allied Health, School of Health Sciences, Swinburne University of Technology, Melbourne, VIC Australia; 2 School of Civil and Environmental Engineering, University of New South Wales, Sydney, NSW Australia; 3 Erasmus School of Health Policy & Management, Erasmus University Rotterdam, Rotterdam, The Netherlands

**Keywords:** Health economics, Cost-effectiveness, Microeconomics, Macroeconomics, Scientometric analysis

## Abstract

**Introduction:**

Health economics is a thriving sub-discipline of economics. Applied health economics research is considered essential in the health care sector and is used extensively by public policy makers. For scholars, it is important to understand the history and status of health economics—when it emerged, the rate of research output, trending topics, and its temporal evolution—to ensure clarity and direction when formulating research questions.

**Methods:**

Nearly 13,000 articles were analysed, which were found in the collective publications of the ten most specialised health economic journals. We explored this literature using patterns of term co-occurrence and document co-citation.

**Results:**

The research output in this field is growing exponentially. Five main research divisions were identified: (i) macroeconomic evaluation, (ii) microeconomic evaluation, (iii) measurement and valuation of outcomes, (iv) monitoring mechanisms (evaluation), and (v) guidance and appraisal. Document co-citation analysis revealed eighteen major research streams and identified variation in the magnitude of activities in each of the streams. A recent emergence of research activities in health economics was seen in the Medicaid Expansion stream. Established research streams that continue to show high levels of activity include Child Health, Health-related Quality of Life (HRQoL) and Cost-effectiveness. Conversely, Patient Preference, Health Care Expenditure and Economic Evaluation are now past their peak of activity in specialised health economic journals. Analysis also identified several streams that emerged in the past but are no longer active.

**Conclusions:**

Health economics is a growing field, yet there is minimal evidence of creation of new research trends. Over the past 10 years, the average rate of annual increase in internationally collaborated publications is almost double that of domestic collaborations (8.4% vs 4.9%), but most of the top scholarly collaborations remain between six countries only.

## Introduction

Health economics, a discipline of economics that focuses on studying how resources are allocated, utilised, and distributed in the healthcare sector [1]. Health economists use various economic tools and techniques, such as cost-effectiveness analysis, cost-benefit analysis, econometric modelling, and microeconomic theory, to examine a wide range of healthcare issues [2, 3]. The field has experienced rapid evolution, largely due to the decades of work of committed scholars. These scholars have not only built a foundation of knowledge, but also developed and refined a set of methodological tools to guide decision making by health care authorities [4]. Modern day health systems are constantly challenged by scarcity of resources, which is attributable to an aging population, diseases of prosperity, rapid urbanisation, technological advancement in the medical field and large scale migrations [4], not to mention the new threat of global pandemics [5, 6]. Another contemporary issue is the rising out-of-pocket health spending that continues to threaten the affordability of medical care, even for some of the most advanced OECD countries [7, 8]. These challenging and complex environments create strong drivers for the further development of health economics.

In 1963, Kenneth Arrow published “Uncertainty and the welfare economics of medical care” in *The American Economic Review* [9]. It became one of the most highly cited articles in health economics and was considered the article that established the field. From here, the term “health economics” increased rapidly in articles published in economics, however, it was not until the early 1980’s that saw the creation of specialised health economics journals.

The unprecedented surge in publications presents researchers with challenges in keeping up with the latest advancements in the field of health economics. Hence, consolidating research and its outcomes has gained even greater importance [10]. For scholars, it is important to understand the history and status of health economics—when it emerged, the rate of research output, trending topics, and its temporal evolution—to ensure clarity and direction when formulating research questions. The course of health economics has been charted previously [11, 12], however, these analyses focus on bibliometric properties of the field. Whilst this is important to report, this paper will extend current knowledge by completing a scientometric analysis of contemporary health economics, using specialised sources and advanced analytical and clustering tools. In health economics, systematic reviews are considered the gold standard for measuring efficacy and effectiveness of a specific topic due to their rigorous nature. However, scientometrics can be utilised to complement systematic reviews to summarise the *overall trends* observed with a topic [10, 13].

The main objectives of our study presented in this paper are to determine the patterns in regional distribution of relevant health economics publications, prominent author networks, the major divisions and research streams of health economics literature, and the variation of activity for each sub-area. This paper also reports on the trending topics and highlights, based on a multitude of objective metrics, the influential references of health economics literature that have shaped the formation of each research stream.

## Methods

### The dataset of references

To retrieve the data for this study, the Web of Science (WoS) Core Collection was accessed and searched in May 2022. A search query was formulated in consultation with an experienced health economist. The ten sources (i.e. scientific peer-reviewed journals) that predominantly publish articles relevant to health economics were included. A list of sources was initially identified if they were listed by the WoS in both categories of “Health Policy & Services” and “Economics”. From this list, the ten sources with the largest volume of content were selected for inclusion in the search. Keywords were not utilised in the search strategy due to the diversity of the terms being used across health economics along with the lack of distinctiveness across other fields (e.g. economics and medicine).

### Search strategy

SO = (“Value in Health” OR “Health Economics” OR “Pharmacoeconomics” OR “Pharmacoeconomics Open” OR “International Journal of Health Economics and Management” OR “Journal of Health Economics” OR “Health Economics Review” OR “Applied Health Economics and Health Policy” OR “American Journal of Health Economics” OR “European Journal of Health Economics”).

Upon initial inspection of the 68,000 documents found by the search strategy, *Value in Health* journal has indexed 54,000 documents as meeting abstracts. These records did not display abstract or reference lists, which are essential for scientometric analysis. Hence, it was determined that for this analysis the inclusion criteria needed to be refined to articles and review articles only. No restrictions were set on other subcategories. The maximum year was set to December 31, 2021, with no restriction on the minimum. Full bibliographic details of the documents were exported from WoS as text files. Details include document title, authors, author affiliations, year of publication, source (journal) title, citation count, document type, abstract, author keywords, keywords plus, funding source, full list of document references and conference information, if relevant.

### Analyses

#### General findings

The estimated size of the literature, highly cited documents, prominent sources and author affiliations (i.e. country and institution) were analysed using the meta data extracted directly from WoS.

#### Semantic analysis

Title and abstract, and keyword analyses were conducted using VOSviewer 1.6.15. Keywords provide insight into the temporal shifts in research and scholarly focus. Clusters of terms extracted from the titles and abstracts are formed by the frequency they occur (set to a minimum of 15) in the articles to provide an objective overview of the structure and divisions within this research topic.

#### Networks of author collaboration

Analyses of author networks were conducted using VOSviewer 1.6.15. Each author is represented by a node and is connected to other authors via links. The number of co-authored documents is indicated by the thickness of the link between the two nodes.

#### Influential articles analysis

Document co-citation and citation burst analyses was completed using CiteSpace 5.7.R1 [14]. The concept of document co-citation, a methodology developed by Chen [15], was used to obtain an indication of the most influential studies within the field of health economics as well as the clusters of thematically similar references. The methodology identifies cohorts of references that are frequently co-cited in the reference lists of health economics papers, on the premise that such references are similar in subjects and represent the knowledge foundation of a certain topic in the field. Document co-citation analysis results in a new set of documents, which include valuable knowledge sources for health economics that are instrumental in the development of this literature but were not captured by the WoS search query.

From document co-citation we can find (i) references with the most local citations (citations from within the literature exclusively relevant to this topic), (ii) references with the strongest citation burst (heightened attention to an individual article within the field, representing a temporal component of the research topic) and, iii) references with the highest centrality (document co-citation across multiple clusters).

#### Temporal analysis

CiteSpace 5.7.R1 [14] was used to generate the dynamic visualisation, which shows insight into the emergence and activities of each research stream since 1990. Research streams are named using the titles of the citing articles (of each stream). Nouns and noun phrases are extracted from the titles. These nouns and noun phrases are each allocated a score depending on the frequency of appearance and the coverage of the citing article they are extracted from (coverage of a citing article refers to the number of cited references of the cluster that it cites). Heavier weighting is given to the noun phrases extracted from high coverage articles because they are more instrumental in the development of the cluster. These noun phrases are sorted based on this score and the top ones are used as a guide for the naming of the cluster. This means that labelling is done by the field expert but guided by an algorithmic determination. In the visualisation, parts of the network that have been most active during each year appear more striking, representing co-citation instances during that year. Influential references are identified using the three metrics (local citations, bursts, centrality). However, these metrics are measuring articles that may or may not be about health economics, so we must also look at the citing articles with the highest *coverage* to determine which articles related to health economics are citing the most references within the specific research stream.

The time period for the analysis was set for 1990–2021 (1-year intervals; look back years = 50 [reference lists published less than 50 years ago]). Each node represents an individual reference. The size of the node is proportional to the number of local citations identified to that reference, and the nodes are connected by links (indicating co-occurrence of co-citation) to create a network of major research streams, all contained within the field of health economics. Each stream has a descriptor based on the contents of the cluster. Furthermore, CiteSpace analysis also provides a timeline view of the evolution of research streams. The references of each stream are visualised and aligned across the timeline based on the year of publication from 1950–2021.

## Results

### General findings and the history of health economics

**Fig. 1:**
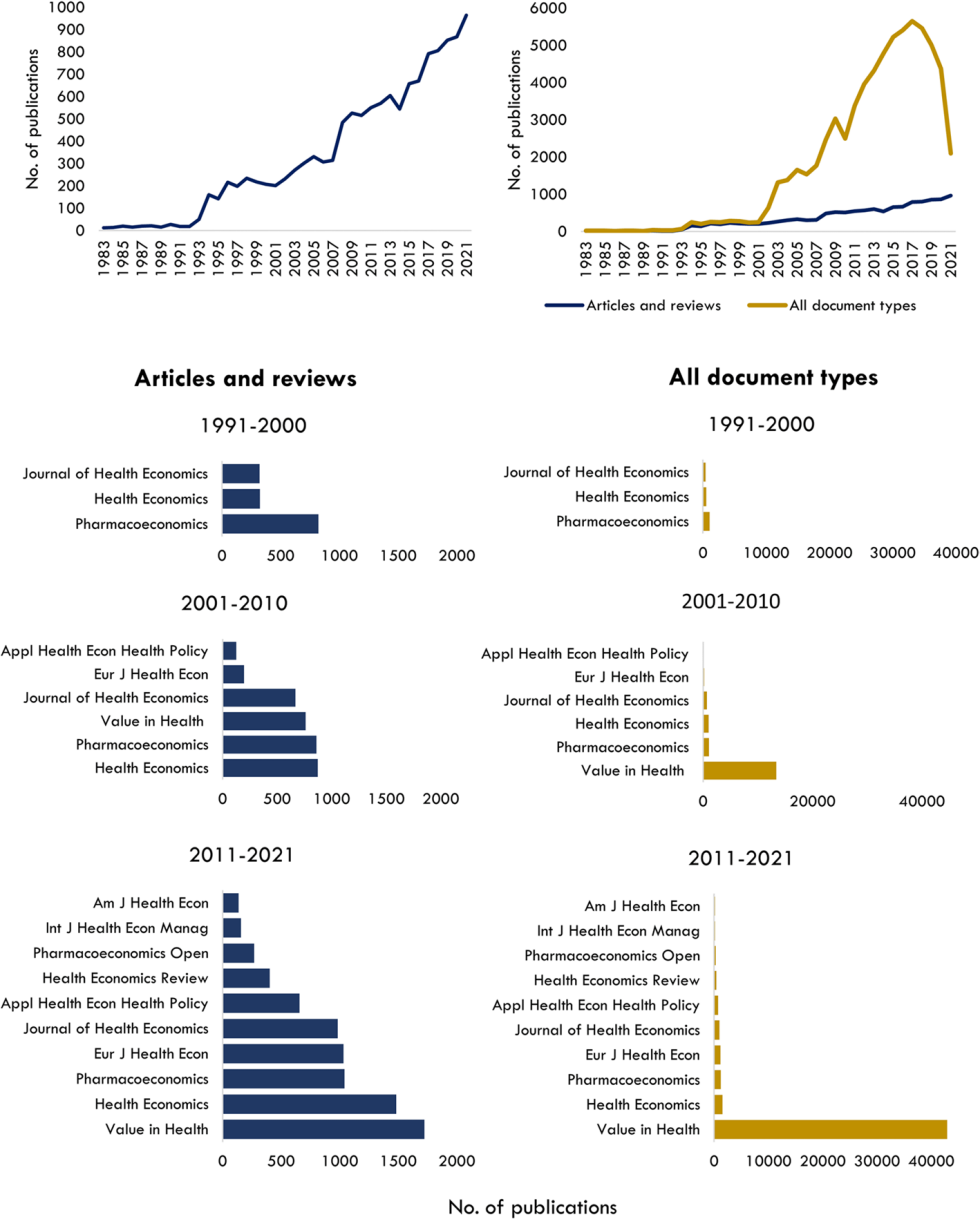
Above (L) Total number of articles and review articles in health economics specialty journals; Above (R) All document types versus total number of articles and reviews in health economics specialty journals; Bar graphs (L) Number of documents by journal source for articles and review articles. Bar graphs (R) Number of documents by journal source for all document types

The size of the specialised field of health economics is estimated to be 12,977 items, as of December 31, 2021. The first article published in a specialty journal (*Journal of Health Economics*) is ‘Effects of teaching on hospital costs’ in 1983 [16]. The following decade saw only a small number of documents published before a significant increase in research output was observed around the mid-1990s (Fig. 1). Since then, there has been an upwards trend, with post-2005 showing a sharp incline in the number of publications.

If all document types were included in the field analysis, there would be nearly 70,000 items, with meeting abstracts published in Value in Health contributing to around 80% of documents (Fig. 1). Over the past three decades, the number of specialised health economics journals in this field has grown from three to ten, with *Health Economics* and *Value in Health* publishing the most literature in 2010–2021 (Fig. 1).

The onset of Covid-19 in early 2020 has not dampened publication of health economics articles and reviews, however, surprisingly only 72 published articles directly explore the topics related to the pandemic. Conversely, a large decline in meeting abstracts has occurred over the past 3 years, however, if and how the pandemic has contributed is unclear, as the decline started in 2019 (from 4,500 to 4000 in the years 18–19) and cannot be solely attributed to a reduction in organised conferences.

An overview of the articles specific subject areas was identified using WoS *Categories*. Unsurprisingly, all records are indexed in the disciplines of Economics and Health Policy Services (12,977 records, 100%). Other categories include Health Care Sciences Services (11,039 records, 85%), Pharmacology Pharmacy (2,992 records, 23%) and Business Finance (156 records, 1%).

Over 26,000 scholars have contributed to health economics research, of which 242 authors have published 15 or more documents related to this field. The top published authors include John Brazier (*n* = 78 records), Werner Brouwer (*n* = 64), Michael Drummond (*n* = 55) and Maarten Postma (*n* = 54). The top ranked academic institutions include the League of European Research Universities (7.5% of total publications), Erasmus University Rotterdam (5%), University of London (5%), University of York [UK] (4.5%) and Harvard University (3.5%).

**Fig. 2:**
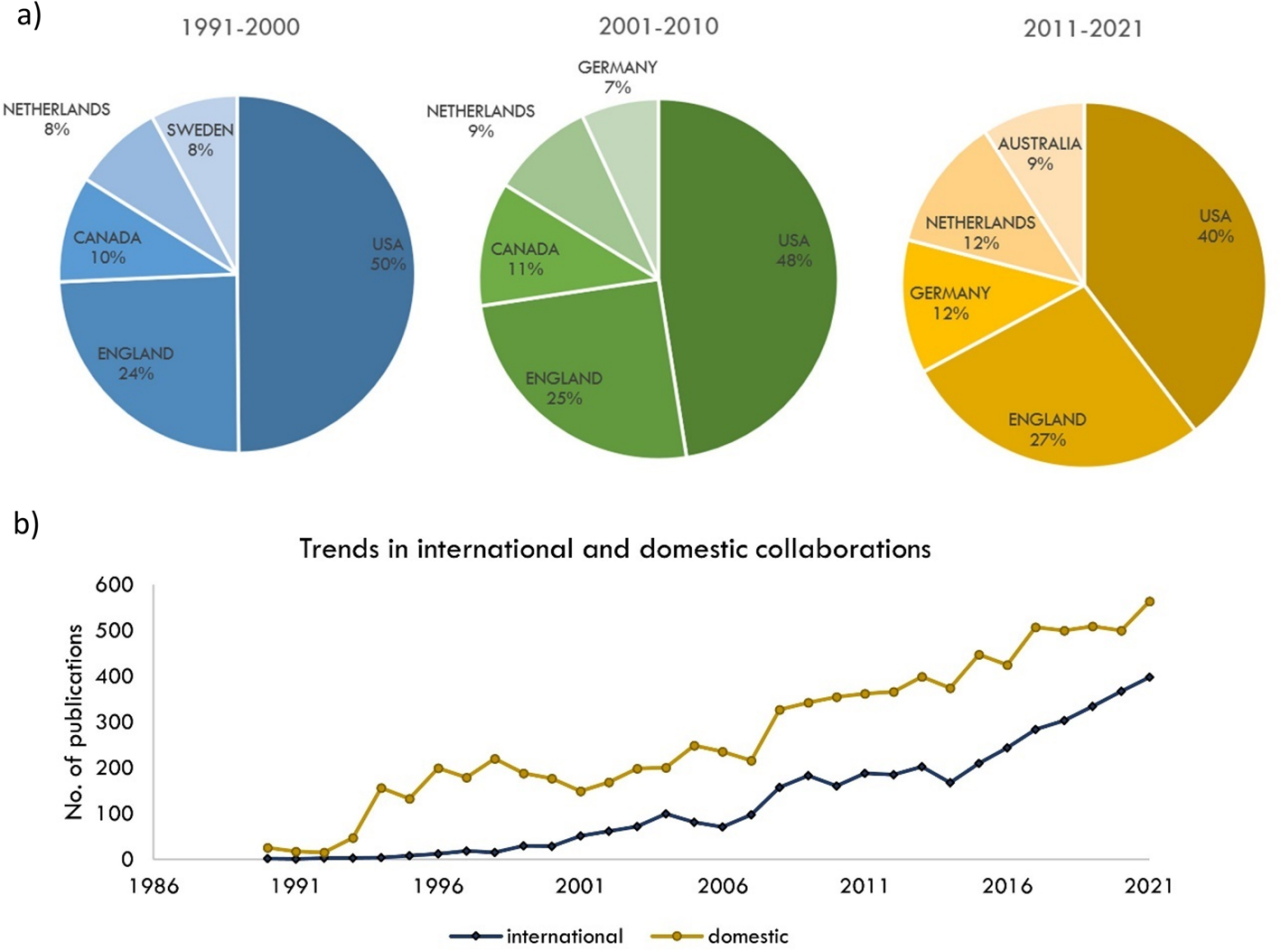
**a** Top five countries to contribute to health economics research output, by decade; **b** domestic versus international collaboration

The main body of research output in health economics is exclusive to six countries: USA, England, Netherlands, Canada, Australia, and Germany. More recently however, countries in Eastern Europe, Africa, Southeast Asia and the Middle East have become more prominent researchers in health economics. Over the previous three decades, the top five countries have remained mostly consistent (Fig. 2), except for Australia, where scholarly output in this area is growing extensively.

Since 2015, international collaboration has been sharply on the rise (Fig. 2). The gap between domestic and international collaborated publications appears to be closing. Currently, domestic publications contribute to 58.6% of the scholarly output compared to 41.4% international publications, however, over the past 10 years, the average rate of annual increase in internationally collaborated publications is almost double that of domestic collaborations (8.4% vs 4.9%). The main six countries in health economics show patterns of strong international collaboration. Together, they have produced approximately one third of the research field (4,000 articles). The strongest links are between the USA and England, USA and Canada, and England and The Netherlands.

### Semantic analysis; titles, abstracts and keywords

**Fig. 3:**
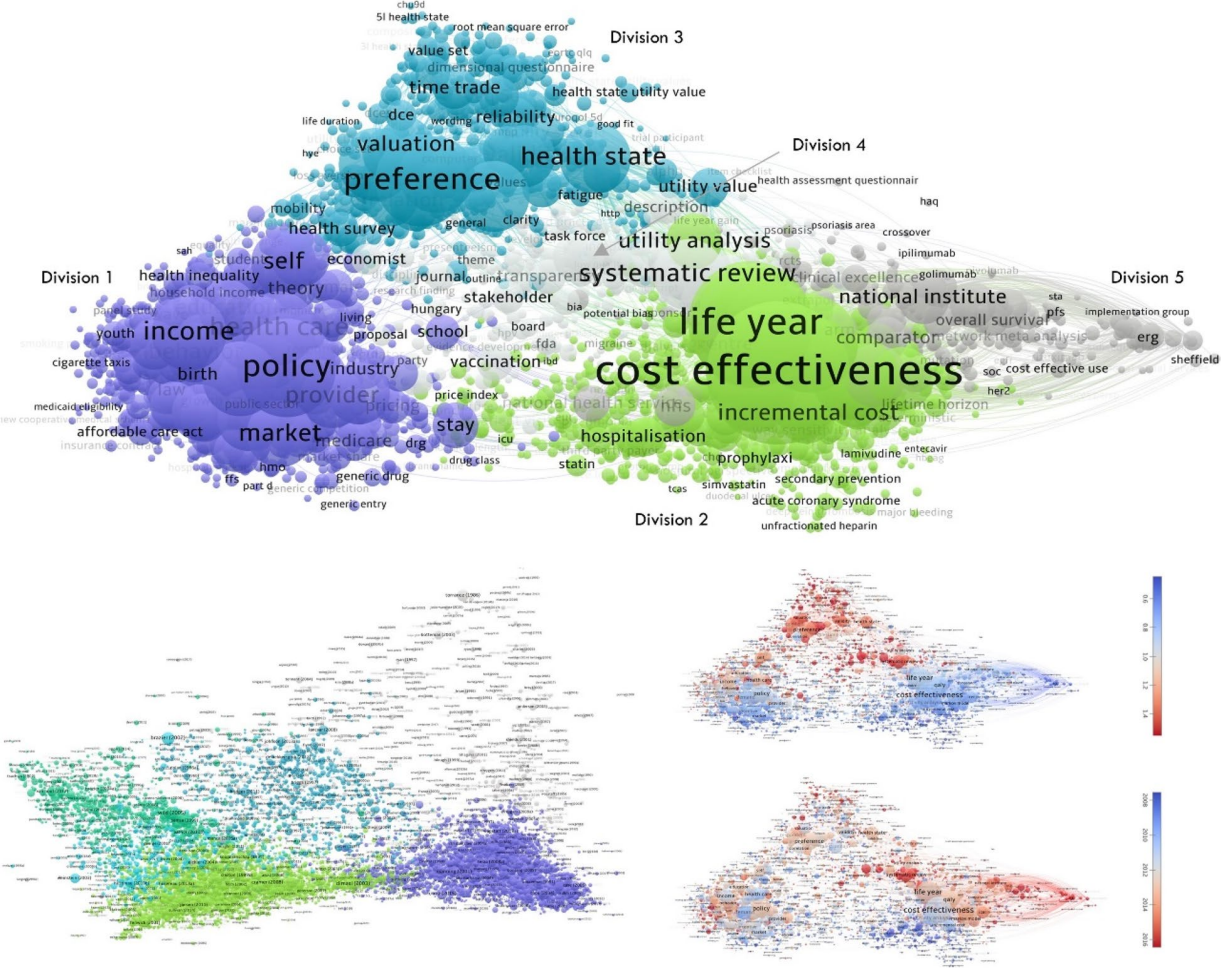
Major divisions of health economics. Below (L) divisions of bibliographic coupling; Below (R) average number of citations and average year of publication for each major division. Interactive version of the title and abstract map are available via this link: VOSviewer Online

Five major divisions were identified in the field of health economics (Fig. 3). 1) Macro-economics, 2) Micro-economics, 3) Measurement and valuation of outcomes, 4) Monitoring mechanisms and 5) Guidance and appraisal. Division 3, measurements and valuation of outcomes is the most cited, and division 5, Guidance and appraisal has the most recent publications.

Bibliographic coupling resulted in similar divisions of health economics research areas. Macro-economics (purple) and micro-economics (green) are the densest divisions, showing extensive overlap of references. Methods for measurement and valuation of patient outcomes, including Discrete Choice Experiments (DCEs), and the EQ-5Dto a lesser extent, are central to both macro- and micro-economics. Table 1 shows the top title and abstract terms of each major division in health economics.

**Table 1:** Top title and abstract terms in each major division

**Division**	**No. of items**	**Top terms**	**Youngest terms**	**Most cited terms**
**1. Macro-economic evaluation (dark blue)**	1412	Policy	Percentage point	Endogeneity
		Health care	Causal effect	Poor health
		Demand	Pandemic	Income group
		Income	Spillover/ spillover effect	Economic growth
		Market	Medicaid expansion	Economic analysis
		Self	Affordable care act (ACA)	National longitudinal survey
		Incentive	Exogenous valuation	Behavioural risk factor surveillance
		Provider	Universal health coverage	Binge drinking
		Utilization	Public health insurance	Job loss
		Behaviour	Channel	Social network
**2. Micro-economic evaluation (green)**	879	Cost-effectiveness	Year time horizon/ lifetime horizon	Long term survival
		Life year	Deterministic	Discontinuation
		QALY/ QALYs	Hazard ratio	Probabilistic analysis
		Incremental cost	Scenario analysis	Randomised clinical trial
		Trial	State transition model	Cost-effectiveness acceptability curve
		Sensitivity analysis	Hepatitis C Virus/ HCV	Ulcer
		Markov model	Model input	Mixed treatment comparison
		ICER	Patients	Natural history
		Utility analysis	T2DM	Individual patient data
		Time horizon	Credible interval	Additional QALY
**3. Measurement and valuation of outcomes (light blue)**	594	Preference	Discrete choice experiment/ DCE/ DCEs	Standard gamble
		Health state	Value set	EQ-5D
		Validity	Dimensional questionnaire	SF-6D
		Respondent	Level EuroQol/ EQ-5D-3L	Descriptive system
		Valuation	Generic preference	Experimental design
		Questionnaire	Predictive accuracy	Functioning
		Instrument	Data quality	Content validity
		Discrete choice experiment	Map/ mapping	Approximation
		Dimension	Health state utility value	Health state utility
		Item	Severity level	Index value
**4. Monitoring mechanisms (evaluation) (light grey)**	**391**	Systematic review	Systematic review	Outcomes research
		Appraisal	Health technology assessment/ HTA	Reporting
		Health technology assessment	Econlit	Checklist
		Guidance	Stakeholder	Statement
		Economic model	Pubmed/ Embase	Good practice
		Transparency	HTA agency	Meeting
		Stakeholder	Website	Pharmacoeconomics
		Description	Hungary	International society/ ISPOR
		Vaccination	Data extraction	Statistical approach
		Medline/ Embase	Cochrane library	Developer
**5. Guidance and appraisal (dark grey)**	**240**	Pound	Care excellence	Critique
		National Institute/ NICE	Overall survival	Clinical evidence
		Manufacturer	Network meta-analysis	Indirect treatment comparison
		Company	Progression free survival	Dissemination
		Centre	ERG	Network meta-analysis
		Submission	NICE single technology appraisal	Critical review
		Care excellence	Patient access scheme	Submission
		Overall survival	Independent evidence review group	
		NHS	Cost effectiveness evidence	
		Comparator	Appraisal committee	

**Fig. 4:**
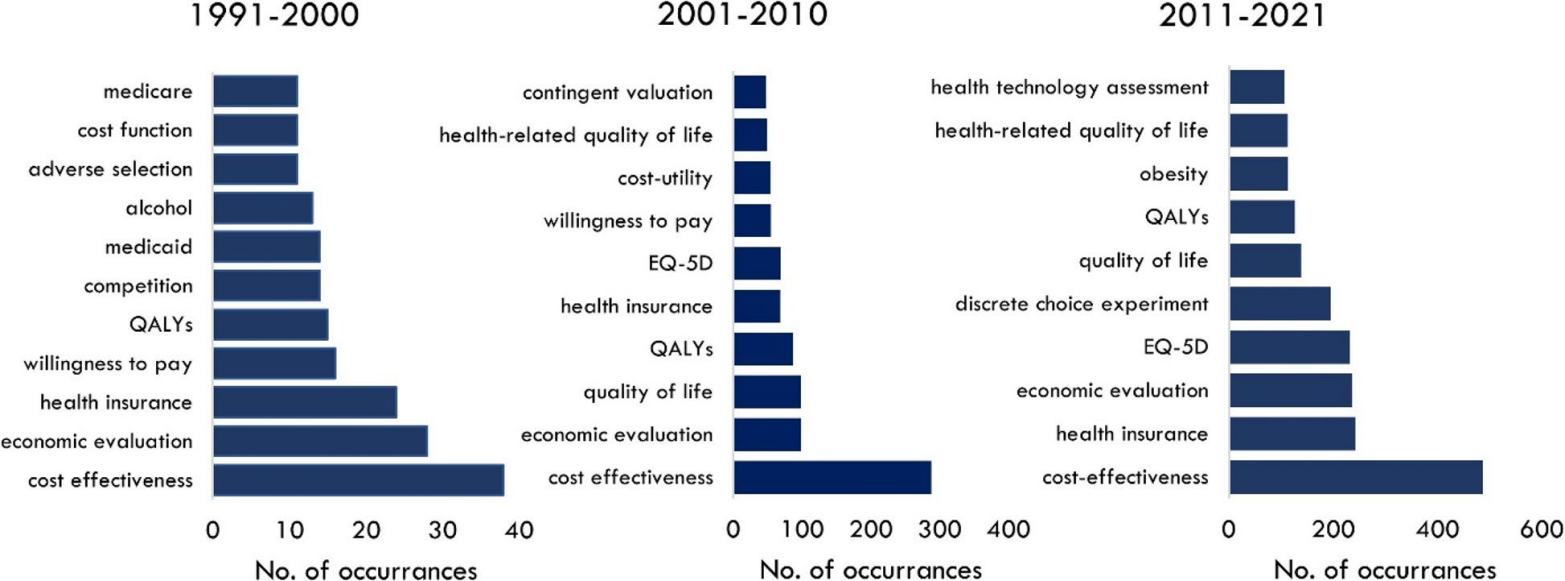
Top keywords in health economics, by decade

The composition of the field of health economics research is dynamic. Keyword analysis across three decades shows there are common research themes including, *cost effectiveness*, *QALYs* and *economic evaluation* (Fig. 4). However, there is a distinct shift to health-related quality of life (HRQoL) in the early millennium, followed by the appearance of DCEs in the most recent decade. Unsurprisingly *obesity*, a global epidemic of the 21^st^ century, has also been a topic of focus for scholarly research since 2010.

### Influential references

This section acknowledges the most influential entities (authors and references) in health economics, aiming to pave the way for further interdisciplinary collaborations and advancements in the domain. These are the most influential entities in a subset of health economics journals. Although the analysis considered a large number of articles (approximately 13,000), it’s important to recognize that there may be other influential entities not represented in this paper.

The top ten globally cited articles have quite distinct topics (Appendix 1). The most cited article, according to WoS, is ‘The price of innovation: new estimates of drug development costs’, authored by DiMasie et al. and is published in Journal of Health Economics. The article has received 2,475 citations, and provides data used to estimate the average pre-tax of new drug development [17].

Influential articles relevant to health economics, ranked by local citation count, are listed in Appendix 2. The most cited article specific to this research field is ‘Recommendations of the Panel on Cost-effectiveness in Health and Medicine’, published in JAMA in 1996 [18]. The authors recommended that if researchers follow a standard set of methods in cost-effectiveness analysis, the utility of studies can be much improved. Lastly, the articles that have had the strongest burst of citations since publication are shown in Appendix 3. This article, published in 2016 and titled ‘Recommendations for Conduct, Methodological Practices, and Reporting of Cost-effectiveness Analyses’ provides major changes to the recommendations made by Weinstein et al. in 1996 [19, 20].

### Temporal analysis

**Fig. 5:**
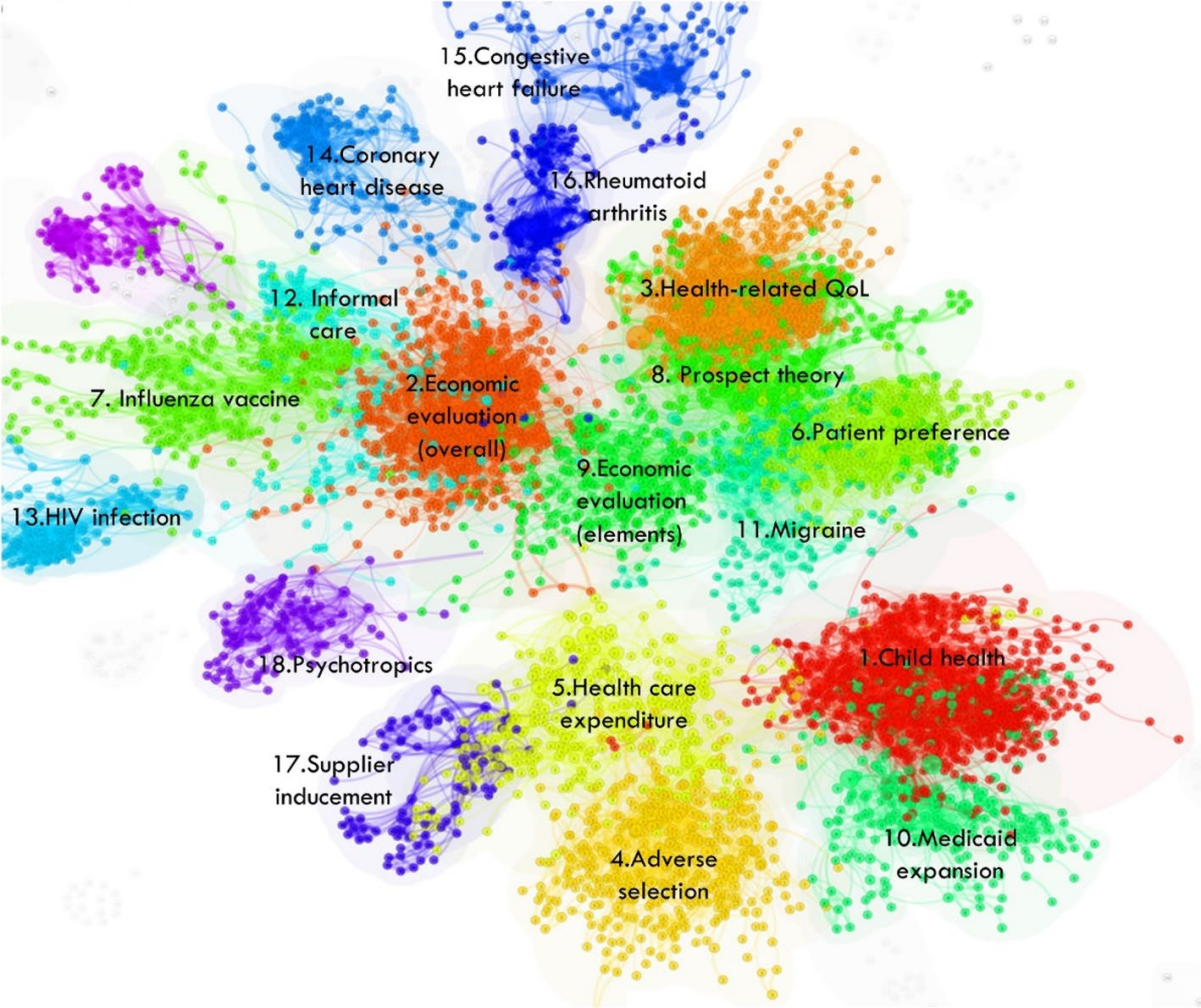
Bird’s-eye view of the major research streams in the field of health economics

**Fig. 6:**
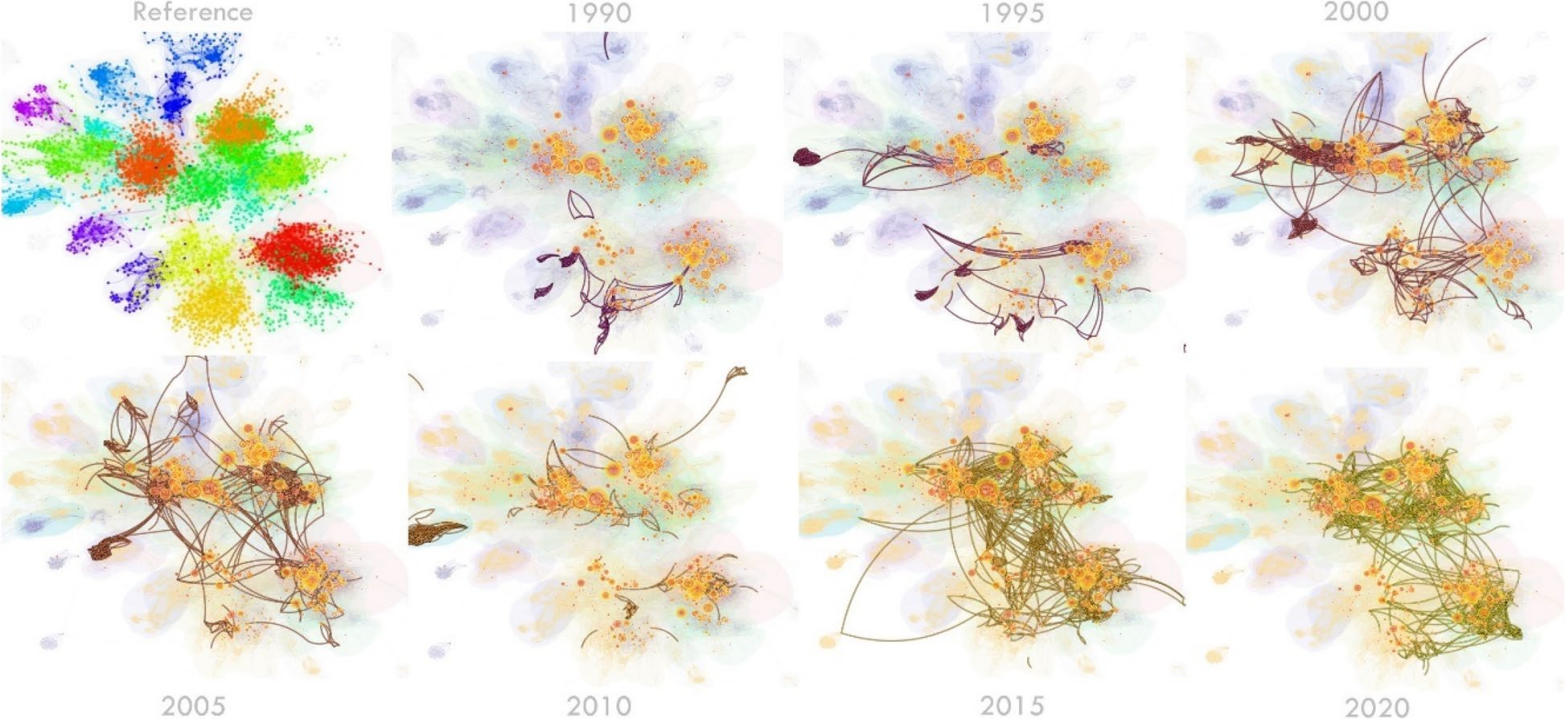
State of health economics literature during the last three decades of development. Salient parts of the map specify active areas of research during each year, as reflected in instances of article co-citation. A dynamic visualisation from 1990–2021 is available here https://unisyd-my.sharepoint.com/:v:/g/personal/clara_zwack_sydney_edu_au/EeT-KZTsqdJHuGzL6s-R9ksBzmQ0ln-2jjYJu5Cv7F0usg?e=pkaOqt

**Fig. 7:**
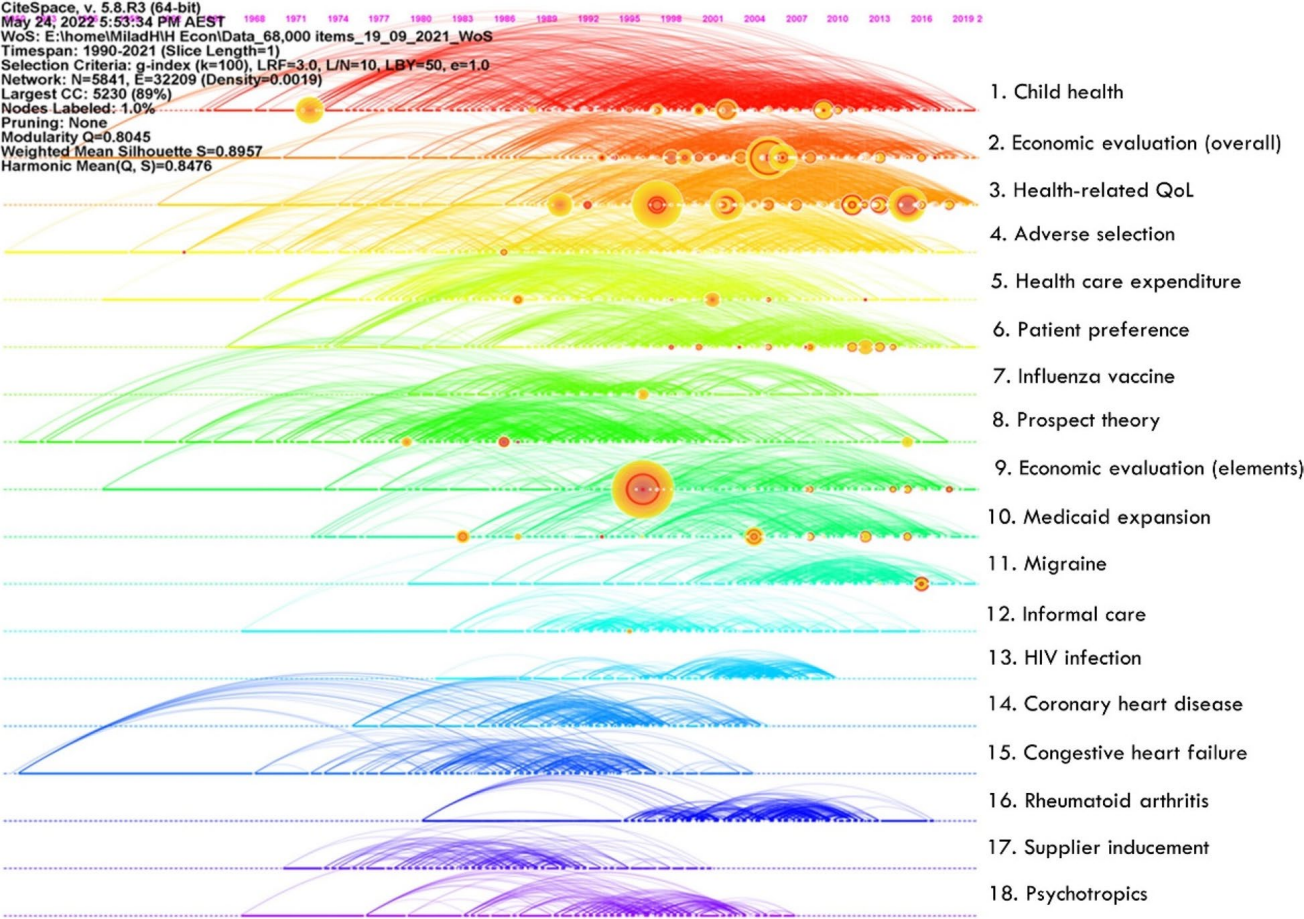
Timeline view of the major research streams in health economics

A major focus is on identifying temporal patterns of scholarly research in this field and the formation of its various research streams as well as the most influential entities within each stream. Document co-citation analysis revealed eighteen research streams. Figure 5 shows a bird’s-eye view of the field and Table 2 identifies the influential references that have shaped each stream. Two streams related to Economic Evaluation emerged, with slight variations. ‘Overall’ Economic Evaluation is broader and includes guidelines, applications of evaluation, reviews of evaluation studies, and articles reporting on willingness to pay studies. ‘Elements’ of Economic Evaluation includes steps involved in evaluation, criteria for evaluation and is mostly focussed on cost-effectiveness studies. These are both central to the field of health economics and are very active areas of research every year, as reflected in instances of article co-citation (Fig. 6). Economic Evaluation is closely related to the activities in Patient Preference and Health-related Quality of Life research (involving measurement tools such as DCEs and EQ-5D, respectively). Figure 7 shows the research streams in time-line format for clear observation of bursts of activity since 1950.

**Table 2:** Major research streams determined by document co-citation output

**Cluster ID** ***Name*** (Author descriptor) **Objective descriptors**	**Cluster statistics** **- size** **- silhouette score** **- mean year (ref)** **- year range (ref)** **- mean year (citing)** **- year range (citing)**		**Influential references**							**Highest coverage citing articles**	
			**Highest local citation count**		**Strongest citation burst (strength, duration)**			**Highest centrality**			
**Cluster #1** ***Child health*** - child health - economic evaluation - systematic review - birth weight - body weight	S	653	[21, 22]	322	[23]^a^	24.82	2005–2014	[24]	0.03	[25]	31
	SS	0.838	[23]^a^	171	[26]^a^	24.68	2014–2021	[27]	0.02	[28]	27
	MY(ref)	2002	[26]^a^	161	[27]	17.54	1999–2007	[29]	0.02	[30]	17
	YR(ref)	1964–2020	[31]	131	[32]^a^	16.18	2017–2021	[31]	0.02	[33]	16
	MY(citing)	2013	[34]	125	[35]^a^	16.14	1990–2011	[36]	0.02	[37]	15
	YR(citing)	1993–2021	[38]	117	[39]	14.39	2014–2018	[21, 22]^a^	0.02	[40]	15
			[24]	81	[41]	12.98	1993–2007	[42]	0.02	[43]	15
			[32]^a^	80	[44]	12.54	2015–2017			[45]	15
			[46]	78	[47]	12.45	2015–2021			[48]	14
			[35]^a^	65	[49]	12.25	2015–2021			[50]	14
			[51]	64		12.16	2001–2010			[52]	14
										[53]	14
**Cluster #2** ***Economic Evaluation (overall)*** -economic evaluation -information analysis -economic model -ispor-smdm modeling -cost effectiveness	S	580	[54]^a^	377	[54]^a^	39.12	2008–2017	[55]^a^	0.03	[56]	39
	SS	0.787	[57]^a^	218		38.01	2010–2014	[58]	0.03	[59–62]	28
	MY(ref)	2005	[63]	151	[64]	30.28	1999–2008	[64]	0.02	[65, 66]	26
	YR(ref)	1954–2020	[67]	144	[67]	24.37	1999–2011		0.02	[68]	24
	MY(citing)	2012	[69]	127	[70]	21.14	2017–2021	[71]	0.02	[65, 66]	22
	YR(citing)	1990–2021	[72]	114		18.96	2006–2011	[69]	0.02	[72]	21
			[64]	109	[73]	18.38	1995–2005	[63]	0.02	[74]	20
			[75]	106	[72]	17.92	2002–2012	[76]	0.02	[77]	18
				101	[59–62]	17.39	2014–2021	[78]	0.02	[79]	17
			[80]	94				[81]	0.02		
**Cluster #3** ***Health-related quality of life*** - health-related quality- achievement goal - 5d-5 l value - preference-based measure - eq-5d health state - economic cost	S	499	[82]	421	[83, 84]^a^	47.63	2015–2021	[85]	0.03	[86]	29
	SS	0.847	[83, 84]^a^	326		41.85	2016–2021	[87]	0.03	[88]	24
	MY(ref)	2006	[89]	271	[90]	38.24	2017–2021	[91]	0.02	[92]	24
	YR(ref)	1961–2020		240		37.06	2016–2021	[93]	0.02	[94]	23
	MY(citing)	2014	[95]	209	[96]	32.34	2018–2021	[97]	0.02	[96]	23
	YR(citing)	1999–2021	[90]	175	[98]	24.96	2016–2021	[99, 100]	0.02	[101]	23
				157	[102]	24.61	2017–2021	[82]	0.02	[103]	23
				142	[59–62]	24.08	2016–2021		0.02	[104]	22
			[85]	133	[105]^a^	20.32	2011–2017	[95]	0.02	[106]	22
			[97]	129				[107]	0.02		
								[108]	0.02		
**Cluster #4** ***Adverse Selection*** - adverse selection - risk selection - moral hazard - economic evaluation	S	379	[109]	97	[109]	16.01	1990–2012	[109]	0.06	[110]	24
	SS	0.926	[111]	81	[112]	12.68	2017–2021			[113]	21
	MY(ref)	1998	[114]	78	[115]	12.06	1998–2007			[116]	15
	YR(ref)	1950–2019	[117]	47	[118]	9.84	2016–2021			[119]	15
	MY(citing)	2011	[120]	42	[121]	9.82	2017–2021			[122]	14
	YR(citing)	1990–2021	[123]	40	[124]^a^	9.77	2015–2018			[125]	14
			[121]	40	[126]^a^	9.6	2017–2021			[127]	14
			[115]	40	[120]	8.84	2015–2021			[128]	13
			[129]	36	[130]	8.05	2018–2021			[131]	13
			[132]	36							
**Cluster #5** ***Health care expenditure*** - health care expenditure - hospital cost - public health expenditure	S	326	[133]	166	[134]	18.46	1990–2009	[135]	0.06	[113]	15
	SS	0.89	[136]	110	[137]^a^	18.42	2012–2020	[134]	0.04	[138]	12
	MY(ref)	1995	[139]	91	[140]	16.86	2000–2010	[139]	0.03	[141]	12
	YR(ref)	1957–2018	[142]	80	[143]	14.29	2010–2015	[144]	0.02		12
	MY(citing)	2008	[140]	77	[145]	12.17	2000–2011	[146]	0.02	[147]	12
	YR(citing)	1990–2020	[144]	74	[144]	12.14	1991–2008	[148]	0.02	[149]	11
			[150]^a^	65	[151]	11.88	1990–2000	[142]	0.02	[152, 153]	10
			[134]	59	[134]	9.69	1994–2005	[154]	0.02	[155]	10
			[145]	59	[156]	8.69	2014–2018			[152, 153]	10
					[157]	8.62	2000–2009			[158]	10
										[159]	10
**Cluster #6** ***Patient preference*** - patient preference - patients preference - using best-worst scaling - contingent valuation studies	S	317	[160]	107	[161]	23.35	2001–2007	[162]	0.02	[163]	30
	SS	0.92	[164]	106	[165]	20.92	2014–2021		0.02	[166]	23
	MY(ref)	2003	[167]	90	[168]	20.85	2016–2021	[169]	0.02	[170]	23
	YR(ref)	1966–2020	[165]	89	[164]	19.72	2013–2021			[171]	19
	MY(citing)	2012	[172]	81	[173]	18.63	2001–2007			[174]	18
	YR(citing)	1996–2021	[169]	76	[175]	16.08	2016–2021			[176]	18
			[161]	66	[177]	16.04	1998–2009			[178]	17
			[179]^a^	64	[180]^a^	15.96	1996–2003			[181]	17
			[182]^a^	60	[183]^a^	15.65	1996–2008			[184]	17
					[182]	15.33	2011–2016				
**Cluster #7** ***Influenza vaccine*** - influenza vaccination - economic evaluation reporting standard - consolidated health - pharmacoeconomic studies - antidepressant prescribing	S	289	[185]	152	[186]	14.3	1996–2005	[187]	0.04	[188, 189]	26
	SS	0.894	[190]	46	[191]	13.21	1997–2001	[192]	0.03	[188, 189]	23
	MY(ref)	1994	[187]	34	[187]	12.15	1995–2002	[185]	0.03	[193]	23
	YR(ref)	1979–2013	[194]	33		11.45	1996–2000	[186]	0.02	[59–62]	18
	MY(citing)	1999	[195]	31	[196]	10.93	1996–2003	[197]	0.02	[59–62]	18
	YR(citing)	1994–2019	[186]	29	[198]	10.84	1996–2000			[59–62]	18
			[191]	22		10.04	1994–1998			[59–62]	18
			[199]	21	[200]	9.87	1997–2000			[201]	18
			[196]	20	[202]	9.25	1997–2000			[200]	17
										[203]	17
**Cluster #8** ***Prospect theory*** - prospect theory - person trade-off - internal consistency - individual preference - standard gamble	S	286	[204, 205]	166	[83, 84]^a^	42.4	2017–2021	[206]	0.03	[207]	33
	SS	0.886	[208]	125	[209]^a^	31.16	1992–1999	[208]	0.03	[210]	30
	MY(ref)	1991	[83, 84]^a^	105	[204, 205]	23.23	1991–2009	[211]	0.03	[212]	24
	YR(ref)	1951–2018	[209]^a^	61	[213]	16.07	1992–2005	[204, 205]	0.02	[214]	18
	MY(citing)	2004	[215]	59	[216]	14.75	1999–2005	[99, 100]	0.02	[217]	18
	YR(citing)	1991–2020	[218]	52	[218]	14.6	1997–2005			[219]	18
			[216]	51	[220]	12.85	1999–2009			[221]	16
			[213]	47	[222]	12.31	1997–2006			[223]	16
			[99, 100]	44	[224]	11.58	1992–1998			[225]	16
			[220]	43	[226]	11.02	1992–2000				
**Cluster #9** ***Economic Evaluation (elements)*** - cost-effectiveness threshold - cost-effectiveness analysis - life year - ispor special task force report - stroke prevention	S	256	[227]^a^	584	[227]^a^	40.93	1998–2009	[217]	0.05	[228]	23
	SS	0.89	[229]	96	[230]	26.61	2016–2021	[229]	0.02	[231]	20
	MY(ref)	2007	[230]	81	[229]	24.63	1994–2007	[232]	0.02	[233]	12
	YR(ref)	1957–2020	[232]	81	[234]	20.6	2018–2021	[235]	0.02	[236]	12
	MY(citing)	2013	[237]	77		19.35	2010–2014			[235]	11
	YR(citing)	1992–2021	[238]	70	[239]	17.07	2016–2021			[240]	11
			[241]	67	[242]^a^	13.93	2017–2021			[243]	11
			[244]	61	[241]	13.19	1998–2008			[234]	10
			[245]	57	[246]	13.02	2007–2012			[247]	9
			[248]	57	[248]	12.53	2006–2013				
**Cluster #10** ***Medicaid expansion*** - medicaid expansion - affordable care act - health insurance - economic evaluation - health insurance coverage	S	252	[249]	188	[250]	23.54	2015–2021	[251]	0.04	[28]	18
	SS	0.923	[251]	160	[252]	20.36	2016–2021			[253]	15
	MY(ref)	2006	[254]	117	[255]	14.56	2014–2021			[256]	15
	YR(ref)	1972–2020	[250]	85	[257]	12.92	2015–2021			[258]	14
	MY(citing)	2016		63	[259]	11.89	2015–2021			[260]	13
	YR(citing)	1990–2021	[252]	62	[261]	11.62	2015–2021			[262]	13
			[255]	62	[263]	11.6	2015–2018			[264]	12
			[265]	52	[266]	11.59	2017–2021			[267]	12
			[268]	50							
			[257]	50							
**Cluster #11** ***Migraine*** - informal care - long-term care insurance - autism spectrum disorder - life outcome - formal care	S	169	[19, 20]	128	[19, 20]	49.8	2018–2021	[269]	0.02	[270]	23
	SS	0.915	[271]	48	[272]^a^	9.56	2002–2009	[271]	0.02	[273]	21
	MY(ref)	2008		46		9.5	2017–2017			[274]	18
	YR(ref)	1979–2020	[275]	41	[276]	9.11	2015–2017			[92]	18
	MY(citing)	2016	[276]	36	[277]	8.69	2008–2016			[170]	18
	YR(citing)	2007–2021	[278]	36	[279]	8.57	2015–2021			[231]	17
			[280]	31	[281]	7.85	2015–2021			[94]	16
			[272]^a^	31	[278]	7.69	2015–2018			[282]	15
			[279]	31							
			[277]	31							
**Cluster #12** ***Informal care*** - multinational investigation - subcutaneous sumatriptan - workplace productivity - osteoporotic fracture - hypertensive patient	S	161	[283]	112	[284]	10.7	2009–2014	[285]	0.02	[286]	19
	SS	0.939	[287]	26		9.45	1998–2004	[287]	0.02	[288]	18
	MY(ref)	1998	[284]	25		8.45	2005–2011	[283]	0.02	[289]	17
	YR(ref)	1967–2016	[290]	20	[291]	7.94	2000–2005			[292]	14
	MY(citing)	2008	[293]	20	[285]	7.21	1996–2003			[294]	14
	YR(citing)	1997–2021	[295]	19	[296]	6.97	1997–2004			[297]	11
				19	[295]	6.91	2007–2014			[298]	10
				17	[299]	6.89	2007–2013			[300]	10
			[285]	17	[287]	6.53	2000–2013			[301]	10
**Cluster #13** ***HIV infection*** - mg bid - treatment-experienced hiv-infected adult - hiv infection - belgium italy sweden - 1-infected adult	S	117	[302]	18	[302]	9.56	1994–1999	[303]	0.04	[304, 305]	52
	SS	0.987	[303]	12	[306]	5.16	2006–2010	[302]	0.03	[304, 305]	52
	MY(ref)	2001	[307]	11	[308]	4.55	1996–2000			[309]	43
	YR(ref)	1981–2010	[308]	11						[310, 311]	42
	MY(citing)	2006	[306]	10						[312]	25
	YR(citing)	1998–2010	[313]	10						[314]	22
			[315]	9						[310, 311]	21
			[316]	9						[317]	19
**Cluster #14** ***Coronary heart disease*** - coronary heart disease - hmg-coa reductase inhibitor - treating dyslipidaemia - using national cholesterol education program - lipid management protocol	S	113		28	[318]	13.7	1997–2005			[319]	28
	SS	0.957	[318]	27		12.12	1997–2005			[320]	25
	MY(ref)	1995	[321]	17	[321]	8.62	1997–2005			[322]	25
	YR(ref)	1975–2005	[323]	16	[323]	7.98	1998–2006			[324]	24
	MY(citing)	2002	[325]	14	[326]	6.85	1998–2005			[327]	18
	YR(citing)	1997–2007	[326]	13	[328]	6.3	2005–2008			[329]	17
			[330]	12	[325]	6.19	2003–2010			[331]	14
			[332]	12	[333]	6.12	2005–2007			[334]	14
					[330]	5.99	1998–2006			[335]	14
					[336]	4.82	1997–2004				
					[337]	4.06	1997–2005				
**Cluster #15** ***Congestive heart failure*** - congestive heart failure - of-life evaluation - needs-based quality - life instrument - ace inhibitor	S	108	[338]	23	[338]	12.47	1996–2000			[207]	29
	SS	0.98	[339]	7	[340]	4.34	1996–1999			[341]	20
	MY(ref)	1988	[340]	7		4.34	1996–1999			[342]	11
	YR(ref)	1951–2004		7	[339]	4.03	1999–2004			[343]	10
	MY(citing)	1998	[344]	6	[345]	3.92	1990–1996			[346]	9
	YR(citing)	1991–2004	[345]	6		3.83	1996–1998			[347]	9
			[348]	6	[344]	3.72	1998–2001			[349]	9
				6	[348]	3.68	2001–2004			[350]	8
**Cluster #16** ***Rheumatoid arthritis*** - rheumatoid arthritis - eastern european countries - anti-tumour necrosis factor-alpha drug - economic decision - including adverse drug event	S	101	[351]	25	[351]	9.83	2012–2015			[56]	32
	SS	0.983	[352]	18	[352]	6.25	2004–2010			[353]	30
	MY(ref)	2003	[354]	11	[355]	5.09	2004–2010			[356]	29
	YR(ref)	1980–2017	[357]	11	[76]	4.72	2012–2014			[358]	20
	MY(citing)	2009	[76]	11	[359]	3.84	2004–2008			[360]	18
	YR(citing)	2004–2020	[361]	11						[362]	14
			[355]	11						[363]	14
										[364]	13
**Cluster #17** ***Supplier Inducement*** - supplier inducement - public-health care system - fee descriptor - multilevel discrete choice model - treatment choice	S	88	[365]^a^	14	[366]	5.44	1993–1999	[367]	0.02	[368]	12
	SS	0.977	[369]	13	[370]^a^	5	1994–2002			[371, 372]	10
	MY(ref)	1986	[367]	9	[373]	4.62	1990–2000			[374]	9
	YR(ref)	1970–2001	[370]^a^	9	[368]	4.46	1999–2007			[371, 372]	9
	MY(citing)	1997	[366]	9	[375]	4.45	1998–2000			[375]	8
	YR(citing)	1990–2007	[368]	9	[365]^a^	4.43	1991–2001			[376]	8
			[373]	8	[367]	4.21	1990–2005				
			[377]	8	[378]	3.82	2001–2003				
			[379]	8	[380]	3.65	1994–1999				
					[381]^a^	3.51	2007–2009				
**Cluster #18** ***Psychotropics*** - long-acting risperidone - economic outcome - antipsychotic agent - long-acting risperidone injection - conventional depot formulation	S	87	[382]	28	[382]	9.81	1998–2006	[383]^a^	0.03	[384, 385]	16
	SS	0.985	[386]	18	[386]	7.46	2008–2010	[382]	0.02	[384, 385]	15
	MY(ref)	1996	[383]^a^	13	[383]^a^	7.02	1995–2003			[387]	13
	YR(ref)	1967–2007	[388]	9	[388]	4.93	2004–2008			[389]	13
	MY(citing)	2004	[390]	7	[391]	3.61	1998–2002			[392]	12
	YR(citing)	1998–2008	[393]	6						[394]	12
			[395]	6						[396]	12
			[397]	6						[398]	11
			[391]	6						[399]	11
			[400]	6							

^a^Indicates the reference is a book

**Fig. 8:**
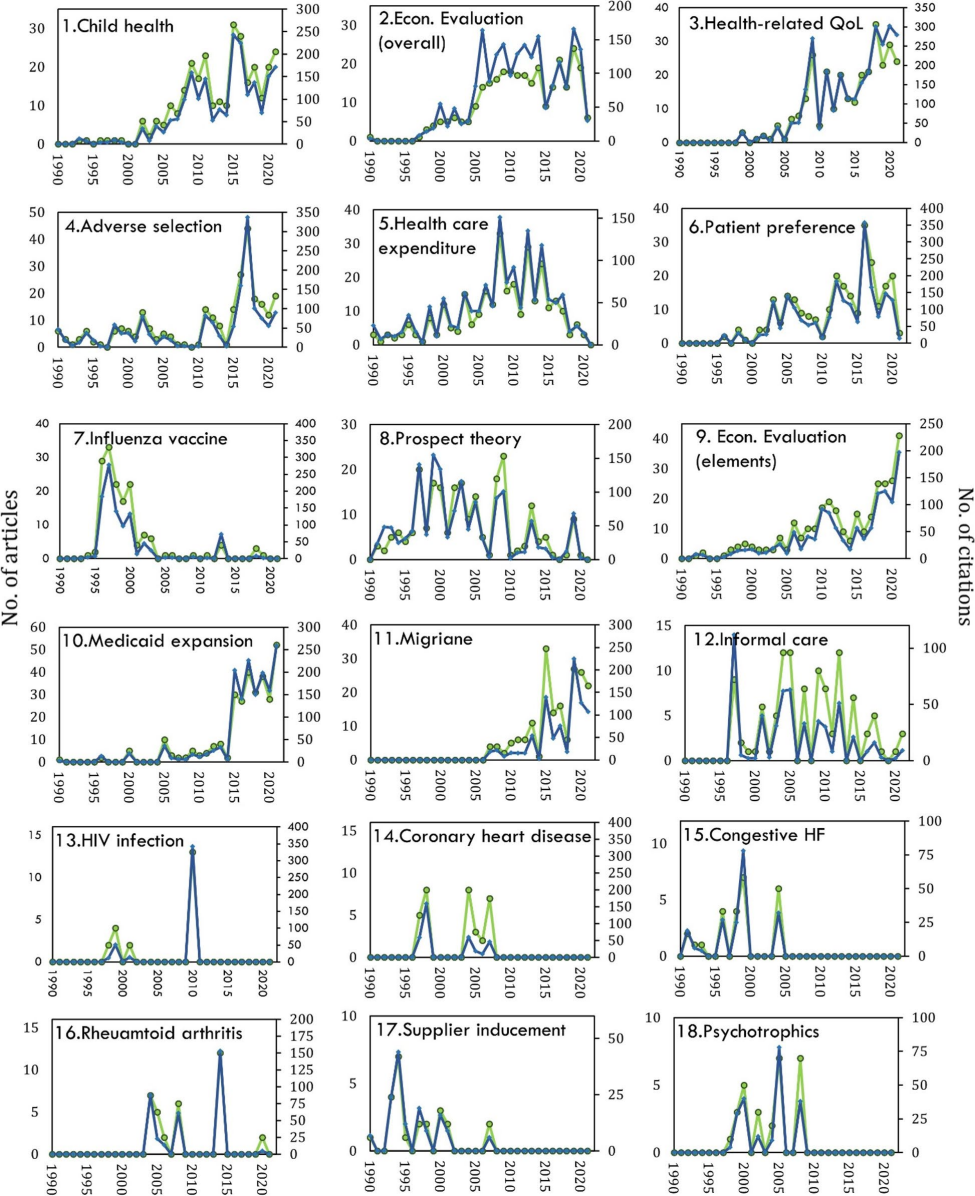
Number of citations (blue) and number of citing articles (green) for each research stream. Note: scale is different for each cluster. Y-axis is Number of articles and X-axis is Number of citations

Co-citation also identified variation in the magnitude of activities in each of the streams (Fig. 8). A recent emergence of heightened research activities in health economics was only seen in the Medicaid Expansion stream. Medicaid expansion is an United States initiative with the goal to increase insurance coverage among low-income adults. It became effective in January 2014, which aligns with the clusters research activity increasing around 2015. Established research streams that continue to show high levels of activity include Child Health, HRQoL and Economic Evaluation (elements). Conversely, Patient Preference, Health care Expenditure and Economic Evaluation (overall) are now past their peak of activity and are slowing down in specialised health economic journals.

Three streams show fluctuating patterns of activity: Adverse Selection (a phenomenon where individuals with higher risks or health issues are more likely to seek or retain health insurance coverage compared to individuals with lower risks), Migraine and Rheumatoid Arthritis. Analysis also identified several streams in this field that have transient peaks of activity and are currently not active. These include Influenza Vaccine, Prospect Theory, Coronary Heart Disease, Congestive Heart Failure, Supplied Inducement and Psychotropics. Lastly, HIV Infection had a very transient period of activity in the early 2000’s. It has since been mostly non-existent, aside from a distinct peak in 2010 where 13 citing articles gave a total coverage of around 140. The critical references were studies measuring the cost effectiveness of Darunavir/Ritonavir, a HIV antiviral drug [305, 309, 311].

## Discussion

This scientometric analysis presents an overview of health economics research exclusively from the top journals specific to the field. Evaluation of around 13,000 documents has revealed contemporary patterns of publication, authorship, and research activities. Five major divisions have been identified within the field using objective clustering methods. This includes macro-economics, micro-economics, measurement and valuation of outcomes, monitoring mechanisms (evaluation), and guidance and appraisal. Along with the major divisions, analysis of document co-citation revealed eighteen specific research streams, each showing varying levels of activity.

Interestingly, there are few ‘hot topics’ emerging in health economics. One possible reason for this could be that the pace of research in health economics could be to some degrees determined by the field of economics and advancement within that mother field, which is considered slow-moving in terms of establishment of new trends [401]. Economists tend to be cautious in recognising emerging areas of research, and instead prefer to use an established knowledge base when supporting their research with previous literature.

In a world where digital transformation is changing the face of every industry, including health care, it is surprising that economic evaluation of digital health innovations has not emerged as a trending research topic. However, there are examples in the literature highlighting the complexities of economic analysis for digital health innovations, which may be stalling the progression of this research area [402–404]. As the knowledge foundation for these freshly emerging areas develop, subsequent analyses of similar nature may be able to detect them as emerging divisions. This knowledge foundation could currently be scattered and not established. The emergence and progression of such area, however, could be detectable with a time lag once the health economics literature begins to converge on a specific cohort of references as the knowledge base in this area.

A sharp rise in scholarly output in health economics was observed around 2005. This is likely around the time that DCEs and patient preference surveys became trendy in healthcare [405]. After heightened research activity in this area for a decade (2005–2015), the Patient Preference research stream has now passed its peak in specialised health economic journals. However, this does not necessarily mean that it is no longer trendy. In fact, it is known that DCEs have now been more widely adopted to elicit preferences for health care products and programs across most medical fields [164, 406]. Peer-reviewed articles are now likely being published in discipline-specific or broader health journals (e.g., British Medical Journal, Health Service Research Journal), rather than the health economics sources used in this analysis.

The main body of this literature has been produced by six countries in Europe, North America and Australia. Since the inception and rapid growth of health economics in the early 1990s, contribution to scholarly literature from these six countries has mostly been consistent, aligning with reports by Wagstaff and Culyer [12]. Few non-OECD countries are included in the top contributors to this research field. For example, China, which now surpassed the USA as the largest producer of scientific research in certain disciplines [407], is not a major contributor to health economics research. However, this may be because China’s primary research foci are technological fields and chemistry, and not social sciences. It is also promising to see recent health economic research output increasing in Low- and Middle-Income Countries. Internationally collaborated research output appears to be moving closer to the domestic output, a promising sign of a connected research field. However, the diversity of health care systems and unique public health issues will likely ensure that domestic research continues to thrive. Applications of new knowledge are often exclusive to a standalone health care system.

It should be noted that the conclusions of this study rely only on a sample of the literature of health economics, by analysing the collective content of ten mainstream health economics journals. While large enough to identify the research trends in the field, as the main motive of the study, the underlying dataset does not necessarily embody the entire literature of health economics. This limitation is simply due to the fact that an attempt for obtaining the entirety of health economics literature seems impossible without jeopardising the dataset with too many false positives. However, it should also be considered that the analytic methodology from which the core findings have been obtained has been chosen such that trends can be identified with minimal sensitivity to missing items in the dataset. The methodology of document co-citation analysis that has produced the core findings of the study is fairly robust to the effects of sampling and potential missing items. This is simply due to the fact that, in this methodology, influential references as well as trends are identified by referring to the reference lists of the articles in the dataset. In other words, the entities of analysis are items listed as the references of the papers in the dataset as opposed to the articles of the dataset itself (as in an article *bibliographic coupling* analysis for example [408, 409]). In a document co-citation approach, the formation of a cluster on topic X does not rely capturing all citing articles that have contributed to the creation of stream/cluster X. If a large enough subset of such citing articles are captured in the data, then stream X as well as its temporal trends will still manifest. This is particularly the case in relation to the major streams (as opposed top smaller/minor clusters) whose sensitivity to the sample is minimal. For that reason, the analyses of this study were limited exclusively to interpreting the major streams only and minor clusters were excluded from an in-depth interpretation. For a typical cluster on a topic such as X, it is possible that papers outside the content of the ten specialty journals (i.e., the current dataset) are also identifiable, in addition to papers related to such topic and disseminated in mainstream specialty journals. But so long as enough of such papers do exist within the content of specialty journals, then the cohort of references co-cited by those papers will still form that stream and topic X along with the temporal patterns of its evolution is still captured by the sample. In summary, the coverage of the underlying data of this study can be improved, but at the same time, we believe that the sensitivity of the main findings to potential missing literature is rather minimal.

## Conclusion

The current state of research in health economics has brought valuable insight into healthcare interventions, market dynamics and behavioural factors. Health economics is a growing field, yet there is minimal evidence of creation of new research trends. This doesn’t necessarily indicate that there are no ‘hot topics’ in health economics, but likely that the new research is being disseminated in sources beyond the speciality journals. Over the past 10 years, the average rate of annual increase in internationally collaborated publications is almost double that of domestic collaborations (8.4% vs 4.9%), but most of the top scholarly collaborations remain between six countries only.

Several avenues for future research exist to deepen our understanding and address the evolving challenges in this field. By considering broader societal perspectives, embracing technological advancements, and integrating behavioural insights, health economist researchers can contribute to evidence-based policy-making and drive improvements in healthcare outcomes, efficiency, and equity.

## Data Availability

All data is available upon reasonable request to the corresponding author.

## Appendix 1

**Table 3:** Most cited papers (WoS dataset)

**Title**	**Authors (year)**	**Journal**	**Citation count (global)**
The price of innovation: new estimates of drug development costs	[17]	Journal of Health Economics	2,475
Principles of good practice for the translation and cultural adaptation process for patient-reported outcomes (PRO) measures: Report of the ISPOR Task Force for Translation and Cultural Adaptation	[410]	Value in Health	2,314
The estimation of a preference-based measure of health from the SF-36	[89]	Journal of Health Economics	2,051
The validity and reproducibility of a work productivity and activity impairment instrument	[411]	Pharmacoeconomics	1,578
Measurement of health state utilities for economic appraisal - a review	[204, 205]	Journal of Health Economics	1,457
Estimating log models: to transform or not to transform?	[133]	Journal of Health Economics	1,515
Medication compliance and persistence: Terminology and definitions	[284]	Value in Health	1,331
Innovation in the pharmaceutical industry: New estimates of R&D costs	[412]	Journal of Health Economics	1,272
The Epidemiology of Obesity: A Big Picture	[413]	Pharmacoeconomics	1,129
Conjoint Analysis Applications in Health-a Checklist: A Report of the ISPOR Good Research Practices for Conjoint Analysis Task Force	[172]	Value in Health	907

## Appendix 2

**Table 4:** Most cited papers by local citation count (document co-citation output)

**Title**	**Authors (year)**	**Journal**	**Citation count (local)**
Recommendations of the Panel on Cost-effectiveness in Health and Medicine	[18]	JAMA	584
Modeling valuations for EuroQol health states	[82]	Medical Care	421
Methods for The Economic Evaluation of Health Care Programmes, 4^th^ Ed^a^	[83, 84]	N/A	326
On the Concept of Health Capital and the Demand for Health	[21, 22]	Journal of Political Economy	322
The estimation of a preference-based measure of health from the SF-36	[89]	Journal of Health Economics	271
EuroQol--a new facility for the measurement of health-related quality of life	[414]	Health Policy	240
Decision Modelling for Health Economic Evaluation^a^	[415]	N/A	218
EuroQol: the current state of play	[95]	Health Policy	209
How Much Should We Trust Differences-In-Differences Estimates?	[249]	The Quarterly Journal of Economics	188
Development and preliminary testing of the new five-level version of EQ-5D (EQ-5D-5L)	[90]	Quality of Life Research	175

^a^Indicates the reference is a book

## Appendix 3

**Table 5:** Articles with strongest bursts of citation in the literature (document co-citation output)

**Title**	**Author(s) (year)**	**Journal**	**Begin**	**End**	**Strength**
Recommendations for Conduct, Methodological Practices, and Reporting of Cost-effectiveness Analyses	[19, 20]	JAMA	2018	2021	49.8
Methods for The Economic Evaluation of Health Care Programmes, 4^th^ Ed^a^	[83, 84]	N/A	2017	2021	42.4
Guide to the Methods of Technology Appraisal 2013	[416]	N/A	2016	2021	41.85
Recommendations of the Panel on Cost-effectiveness in Health and Medicine	[18]	JAMA	1998	2009	40.93
Development and preliminary testing of the new five-level version of EQ-5D (EQ-5D-5L)	[90]	Quality of Life Research	2017	2021	38.24

^a^Indicates the reference is a book
